# Exercise Metabolism in Nonobese Patients with Type 2 Diabetes Following the Acute Restoration of Normoglycaemia

**DOI:** 10.1155/2017/8248725

**Published:** 2017-05-29

**Authors:** Christopher J. Gaffney, Peter Mansell, Francis B. Stephens, Ian A. Macdonald, Kostas Tsintzas

**Affiliations:** ^1^Medical Research Council/Arthritis Research UK Centre for Musculoskeletal Ageing Research, School of Life Sciences, The University of Nottingham Medical School, Queen's Medical Centre, Nottingham NG7 2UH, UK; ^2^School of Sport and Health Sciences, University of Exeter, St Luke's Campus, Exeter EX1 2LU, UK

## Abstract

This study investigated how acute restoration of normoglycaemia affected energy metabolism during exercise in nonobese patients with type 2 diabetes. Six subjects (mean ± SEM) aged 56.2 ± 2.7 years, with a BMI of 24.5 ± 1.5 kg/m^2^ and a VO_2 peak_ of 28.7 ml/kg/min, attended the lab on two randomised occasions for a four-hour resting infusion of insulin or saline, followed by 30 minutes cycling at 50% VO_2 peak_. During the 4 h resting infusion, there was a greater (*P* < 0.0001) reduction in blood glucose in insulin treatment (INS) (from 11.2 ± 0.6 to 5.6 ± 0.1 mmol/l) than in saline treatment/control (CON) (from 11.5 ± 0.7 to 8.5 ± 0.6 mmol/l). This was associated with a lower (*P* < 0.05) resting metabolic rate in INS (3.87 ± 0.17) than in CON (4.39 ± 0.30 kJ/min). During subsequent exercise, blood glucose increased significantly in INS from 5.6 ± 0.1 at 0 min to 6.3 ± 0.3 mmol/l at 30 min (*P* < 0.01), which was accompanied by a lower blood lactate response (*P* < 0.05). Oxygen uptake, rates of substrate utilization, heart rate, and ratings of perceived exertion were not different between trials. Insulin-induced normoglycaemia increased blood glucose during subsequent exercise without altering overall substrate utilization.

## 1. Introduction

Around 90% of adults with type 2 diabetes (T2D) are overweight or obese [1], and a previous research has shown that glycaemic control can be improved in these individuals by weight loss through a structured exercise program or a nutritional intervention [2, 3]. Little research is conducted on the c. 360,000 UK-based nonobese patients with T2D (BMI < 25 kg/m^2^) (based on the data in [4]), which still possess a significant risk of secondary complications [5] and have an equal risk of cardiovascular disease to their obese counterparts [6].

Exercise is an important component in the management of T2D as it can help control weight and improve cardiovascular fitness [7], which are important to mitigate secondary complications. Exercise is also crucial to improving insulin sensitivity [8] and overall glycaemic control [9]. Exercise does, however, present potential challenges in patients with T2D, and this includes the risk of hypoglycaemic episodes, particularly in those taking sulfonylureas and insulin [10]. During exercise, patients with T2D have normal or elevated rates of skeletal muscle glucose disposal but impaired hepatic glucose output [11], increasing the risk of hypoglycaemia. Moreover, exercise increases insulin sensitivity for up to 72 hours postexercise [12], which presents a risk of hypoglycaemia during recovery from exercise. Overweight and obese patients with T2D not taking insulin are able to reduce their blood glucose levels during exercise [13–15], and insulin and exercise synergistically increase muscle glucose uptake [16]. Reductions in blood glucose have also been observed in patients with T2D with both mild [13] and substantial [14, 17] preexercise hyperglycaemia. Both moderate intensity continuous exercise and particularly high intensity exercise are able to reduce nocturnal/fasting glycaemia [15], potentially predisposing to the risk of hypoglycaemia in the fasted state. In addition, plasma glucose utilization is increased during exercise in nonobese [18] and obese [19] patients with T2D. Therefore, patients taking oral glucose-lowering medication prior to exercise are at a heightened risk of hypoglycaemia.

The aim of this study was to determine if exercise in normoglycaemic nonobese patients with T2D was possible without significant reductions in blood glucose levels. Indeed, we sought to determine whether restoration of preexercise normoglycaemia in nonobese patients with T2D was associated with changes in perceived exertion, substrate utilization, or hypoglycemic episodes during subsequent moderate exercise.

## 2. Research Design and Methods

### 2.1. Subjects

Six nonobese and nonsmoking participants (five male) with a diagnosis of type 2 diabetes gave written informed consent to participate in this study, which was approved by Nottingham University Hospital Ethics Committee and conformed to the Declaration of Helsinki. All participants had type 2 diabetes for at least three years (mean duration of 7.8 yrs and range of 3 to 13 years) and had suboptimally controlled diabetes [HbA1c > 8% (64 mmol/mol)]. Subjects were recruited from Nottingham University Hospital Diabetes Register and from participating General Practitioner Surgeries. Subjects were 56.2 ± 2.7 years of age, were 66.7 ± 6.0 kg in weight, and had a BMI of 24.5 ± 1.5 kg/m^2^ and a VO_2 peak_ of 28.7 ml/kg/min. Subjects were excluded if they had significant complications of diabetes, vascular disease, abnormal renal or hepatic function, or other disorders that prevented exercise, including respiratory diseases and arthritis (Table 1). Furthermore, participants were excluded if they produced a recent abnormal Bruce protocol treadmill test to stage III [20]. Subjects were not taking medications that may alter the response to exercise including beta blockers and calcium channel blockers.

### 2.2. Preliminary Measurements

Subjects attended the laboratory prior to the main experimental visits where they were familiarized with the ventilated canopy system (GEM Indirect Calorimetry, GEMNutrition Ltd., Daresbury, UK) that was used for the measurement of a resting metabolic rate [23]. Subjects then completed an incremental exercise test to the maximum predicted heart rate (220 minus age in years) using an electrically braked cycle ergometer (Lode, Groningen, Netherlands) for the determination of maximum oxygen consumption [20]. Before each test, subjects were allowed to warm up at a workload of 20 W for 5 minutes whilst pedaling at 50 rpm. The test used involved a continuous incremental test to exhaustion, with the workload being increased progressively every 3 min by 15–30 W from an initial workload of 40 W. The oxygen uptake during the last minute of the test was taken as the VO_2 peak_ value of the individual. Maximal effort was determined by achievement of a maximal predicted heart rate (±10%), a respiratory exchange ratio (RER) exceeding 1.1, a VE/VCO_2_ ratio exceeding 30, and a plateau of O_2_ consumption, although this was not always present (thus, we report VO_2 peak_). During all experimental visits, an online gas analysis system (Vmax 29, SensorMedics, USA) was used to measure O_2_ consumption, CO_2_ production, and the RER. The measurements during this test were used to calculate the workload required (% of VO_2 peak_) during experimental visits.

### 2.3. Experimental Design

Subjects attended the laboratory on two randomized occasions (morning visits), which were separated by at least two weeks. One experimental visit comprised of an insulin infusion (INS trial), and the other a saline infusion (CON trial) whilst resting for four hours. Subjects were asked to maintain habitual levels of physical activity and a typical isocaloric food intake in the 48 hours before experimental visits. Compliance with the isocaloric diet and typical food intake was assessed through the completion of food diaries. Subjects stopped taking medication for the control of T2D 24 hours before each trial and did not take any habitual morning medication on the day of the trial. Subjects attended the laboratory after an overnight fast, which was defined as the cessation of food and drink other than water from 10.00 pm on the previous evening.

At the beginning of each visit, a resting metabolic rate was measured for 20 minutes by indirect calorimetry using a ventilated canopy connected to an online gas analysis system. Substrate oxidation rates were calculated from the measurements of O_2_ consumption and CO_2_ production using stoichiometric equations [23]. Two retrograde cannulae were then inserted in an antecubital vein and a dorsal hand vein for the infusion of insulin/saline and for blood sampling, respectively. The subject's hand and wrist was kept in a hot air box maintained at 55–60°C throughout the trial to arterialize the blood as previously described [24]. Having arterialized the blood for 10 minutes, a blood sample was then taken for the baseline measurement of whole blood glucose and lactate (determined immediately on the YSI, 2300 Stat autoanalyzer), serum insulin, sodium and potassium, and plasma free fatty acid (FFA) concentrations.

Subjects then rested in a semisupine position for 4 hours whilst receiving an infusion of insulin or 0.9% saline. The infusion of human soluble insulin (Actrapid, Novo Nordisk, Copenhagen, Denmark) was started at a rate of 0.05 mU/kg/hr and varied between 0.05 in hour one and 0.01 mU/kg/hr in hour four of the resting phase (average infusion rate at 1.5 ± 0.2 ml/h) to maintain blood glucose levels at approximately 6 mmol/l. Insulin infusion was stopped before the exercise started. Saline was infused at 2.3 ± 0.1 ml/h. Insulin and saline were administered in a single-blind design. For safety monitoring, arterialized venous blood samples were taken every 15 minutes to measure glucose and lactate concentrations using the glucose oxidase and L-lactate oxidase methods, respectively (YSI, 2300 Stat autoanalyzer, Yellow Springs Instruments, Yellow Springs, USA). Data presented are every one hour for blood glucose and insulin during the resting phase. Additional blood samples were taken every 60 minutes to measure serum insulin using a radioimmunoassay (Diagnostics Products Corporation, Llanberis, Wales, UK), serum sodium and potassium by flame photometry, and plasma FFA using a commercially available kit (NEFA-C test, Wako, Osaka, Japan). The resting metabolic rate was further measured during the last 20 minutes of each hour and after the removal of the ventilated canopy. Brachial artery blood pressure and the heart rate were also determined using an automated Dinamap blood pressure monitor (Dinamap vital signs monitor, GE Healthcare, Chicago, IL). Both insulin and saline infusions were stopped at the end of the four-hour rest period.

Following the four-hour rest period of insulin or saline infusion, subjects then completed the exercise portion of the visit, which comprised of 30 minutes cycling at 50% VO_2 peak_. This exercise intensity and duration is recommended to improve glycaemic control by the American College of Sports Medicine and the American Diabetes Association [11] and therefore represents a suitably challenging exercise stimulus for patients with type 2 diabetes. Every ten minutes throughout exercise, expired air and arterialized venous blood samples were collected, and heart rate and blood pressure were measured. At the same time intervals, the subject's rating of perceived exertion was determined using the Borg scale [25]. Following the completion of exercise, subjects were provided with a meal of mixed macronutrient composition and were observed for 1 hour, after which they were permitted to leave the lab once blood glucose concentrations were stabilized.

### 2.4. Statistics

Data met the criteria for normality (a Gaussian distribution) when tested using a Kolmogorov-Smirnov test with Dallal-Wilkinson-Lille. A two-way repeated-measure analysis of variance (ANOVA) was used to determine if there were statistically significant differences in blood metabolites, metabolic rate and rates of substrate utilization between the insulin and control trials, and over time. Separate two-way ANOVA were completed for the resting and exercise phases, respectively. When a significant main effect was observed, a Holm-Sidak test was used to correct for multiple comparisons and locate differences. Data were analyzed using GraphPad Prism (GraphPad Prism 7.0, GraphPad Software Inc.). Data are presented as means ± SEM, and statistical significance was set at *P* < 0.05.

## 3. Results

### 3.1. Resting Phase

**Figure 1:**
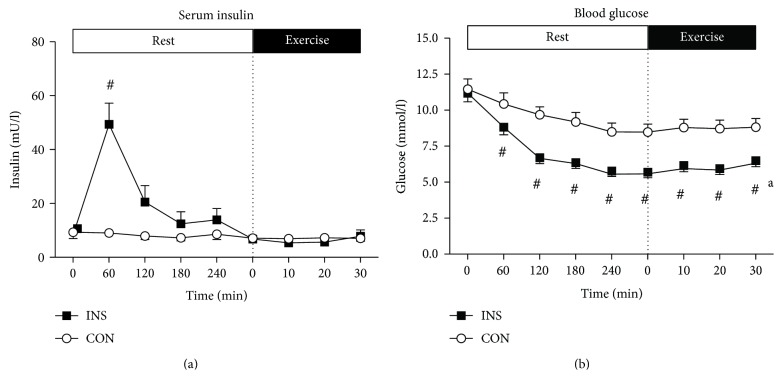
Serum insulin (a) and blood glucose (b) concentrations during the INS and CON trials. Data represent *n* = 6, mean ± SEM. (a) During the resting phase, the infusion significantly increased serum insulin in INS compared to CON (main effect of infusion, *P* < 0.01); there were also significant interaction (*P* < 0.0001) and time (*P* < 0.0001) effects. During exercise, there was no effect of infusion, time, or an interaction. (b) At rest, the infusion significantly lowered blood glucose in INS compared to CON (main effect of infusion, *P* < 0.01); there were also significant interaction (*P* < 0.0001) and time (*P* < 0.0001) effects. During exercise, blood glucose increased significantly in INS (*P* < 0.01) but not in CON. ^#^*P* < 0.0001 from CON; ^a^*P* < 0.01 from immediately before exercise (0 min).

**Figure 2:**
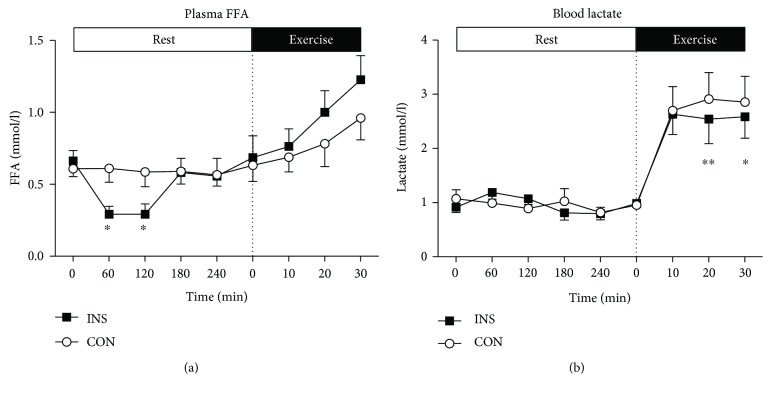
Plasma FFA (a) and blood lactate (b) concentrations during the INS and CON trials. Data represent *n* = 6, mean ± SEM. (a) At rest, there were significant time (*P* < 0.001) and interaction (*P* < 0.05) effects, where plasma FFA were suppressed in INS. During exercise, there was a significant time effect (*P* < 0.001) and a trend (*P* = 0.085) for an interaction effect, where plasma FFA in INS increased to greater extent than that in CON. (b) At rest, there was no significant effect of the insulin infusion or time on blood lactate, although there was a significant interaction effect (*P* < 0.01). During exercise, there were significant time (*P* < 0.001) and interaction (*P* < 0.05) effects, where the blood lactate response was lower in INS than that in CON. ^∗^*P* < 0.05 from CON; ^∗∗^*P* < 0.01 from CON.

In the resting phase, the insulin infusion (INS) significantly increased serum insulin levels in comparison to CON (*P* < 0.0001), peaking after 60 minutes of infusion (Figure 1(a)) and then fell as the insulin infusion rate was lowered in response to the lowering of blood glucose. This higher level of insulin in the INS trial promoted a fall in blood glucose from 11.2 ± 0.6 to 5.6 ± 0.1 mmol/l after 4 h rest in the INS trial, which was lower (*P* < 0.0001) compared with the fall in the CON trial (from 11.5 ± 0.7 to 8.5 ± 0.6 mmol/l, Figure 1(b)). The infusion of insulin suppressed FFA concentrations in the INS trial during the first 2 h of rest compared to CON (*P* < 0.05, Figure 2(a)). The infusion of insulin (INS) was also associated with an increase in average resting RER in comparison to CON (0.93 ± 0.01 versus 0.83 ± 0.02, *P* < 0.05), an increase in average CHO oxidation by 16.6 ± 4.7 g (*P* < 0.05), and a decrease in average fat oxidation by 9.9 ± 2.9 g over four hours (*P* < 0.05, Table 2). During the four-hour infusion period, the average resting metabolic rate was lower in the INS trial (3.87 ± 0.17 kJ/min) when compared to that in the CON trial (4.39 ± 0.30 kJ/min) (*P* < 0.05, Table 2).

### 3.2. Exercise Phase

**Figure 3:**
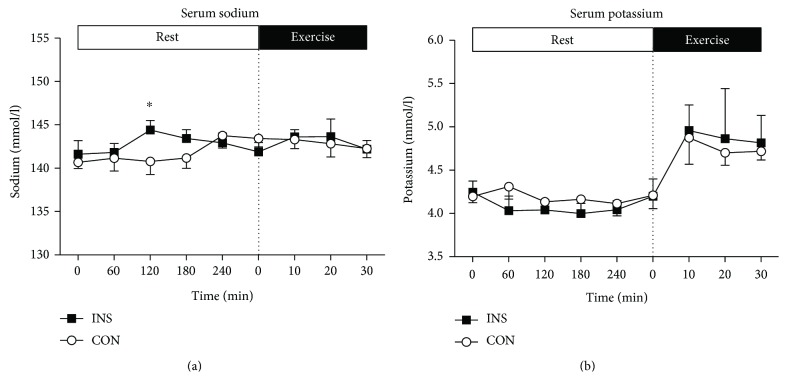
Serum sodium (a) and potassium (b) concentrations during the INS and CON trials. Data represent *n* = 6, mean ± SEM. (a) At rest, the infusion significantly increased serum sodium in INS compared to that in CON (*P* < 0.05) but there were no significant interaction or time effects. During exercise, there were no significant main effects for infusion, time, or interaction. (b) At rest, there was a trend (*P* = 0.060) for lower potassium in INS than in CON and a trend (*P* = 0.063) for time effect. During exercise, there was a significant increase in serum potassium (time effect *P* < 0.05) but there were no significant differences between INS and CON. ^∗^*P* < 0.05 from CON.

Immediately before and during the 30 min of moderate intensity exercise, there were no significant differences in serum insulin levels between INS and CON (Figure 1(a)). Moreover, serum insulin concentrations were maintained at preexercise levels in both groups throughout the exercise phase (INS: 6.3 ± 0.9 mU/l versus CON: 7.0 ± 1.2 mU/l; Figure 1(a)). At the start of exercise, blood glucose was significantly lower (*P* < 0.0001) in the INS trial (5.6 ± 0.1 mmol/l) than in the CON trial (8.5 ± 0.6 mmol/l). During exercise, there was a small but significant increase (*P* < 0.01) in blood glucose in the INS trial (from 5.6 ± 0.1 mmol/l immediately before commencing exercise to 6.3 ± 0.03 mmol/l at 30 min of exercise) (Figure 1(b)). During the same period in the CON trial, blood glucose levels were maintained at 8.8 ± 0.6 mmol/l and were lower than those in INS at all time points (*P* < 0.0001). During exercise, there was a trend (*P* = 0.085) for a greater increase in FFA concentration in the INS trial (from 0.69 ± 0.15 to 1.23 ± 0.17 mmol/l, *P* < 0.0001), when compared with the CON trial (from 0.63 ± 0.11 to 0.96 ± 0.15 mmol/l, *P* < 0.001; Figure 2(a)). There was a greater increase in blood lactate in the CON than in the INS trial (interaction, *P* < 0.05), with higher rates at 20 min (*P* < 0.01) and 30 min (*P* < 0.05) of exercise (Figure 2(b)). There was an increase in serum potassium throughout exercise (Figure 3(b)), with no significant differences between INS and CON trials. There were no significant differences in serum sodium (Figure 3(a)), oxygen uptake, RER, rates of CHO and fat oxidation, heart rate, or ratings of perceived exertion (Table 3) between INS and CON trials.

## 4. Discussion

These data showed for the first time that nonobese patients with T2D display similar metabolic responses to moderate exercise performed in the normoglycaemic and hyperglycaemic states. All subjects in the INS trial completed 30 min exercise without biochemical or symptomatic hypoglycaemia during exercise or the one-hour recovery after exercise, despite starting the exercise phase with a mean blood glucose of 5.6 ± 0.1 mmol/l. As a matter of fact, blood glucose increased during exercise following insulin infusion. Also, following saline infusion, blood glucose did not fall during exercise, suggesting impairment in exercise-mediated glucose disposal in lean subjects with T2D. Blood glucose levels in T2D typically fall due to an increase in skeletal muscle glucose uptake that surpasses any increase in hepatic glucose production [26]. Moreover, since the liver is sensitive to circulating insulin concentrations, the peripheral levels of which were similar between trials, the liver was not likely responsible for this increase in blood glucose. A reduced glucose uptake as a result of reduced mass action effect of glucose was therefore more likely responsible for the absence of lowered blood glucose during exercise. Indeed, blood glucose was lower in INS than in CON throughout the resting phase, which may have led to a lower mass action effect of glucose [27] during exercise. Hepatic glucose production and muscle glucose uptake were not directly measured in this study, however, and therefore, future studies to directly test this hypothesis are warranted. Subcutaneous insulin poses a risk of hypoglycaemia during subsequent exercise, and data in this study are not sufficient to mitigate this concern. Patients on insulin therapy display higher levels of serum insulin [28] than those displayed in this study at the start of exercise. Despite the higher blood glucose in the CON trial during both the resting and exercise phases, RER values were not significantly different between INS and CON during the exercise phase. This suggests a similar contribution of CHO and fat oxidation to energy metabolism.

Patients with T2D and high preexercise blood glucose levels display an increase in peripheral glucose uptake compared to euglycaemic, insulin-sensitive controls [16]. As a result, previous studies with both mild [13] and high [14, 17] pre-exercise hyperglycaemia have shown reductions in blood glucose during exercise. Other studies, however, similar to the present study, have not shown a reduction in blood glucose levels during exercise in the fasted state [19], perhaps due to the insulin resistance associated with an overnight fast [29]. As far as we are aware, the present study is the first in which no lowering in blood glucose was observed in nonobese patients with type 2 diabetes during exercise performed under normoglycaemic conditions.

There was a tendency for a greater increase in plasma FFA concentration during exercise in INS compared with that in CON. Greater levels of plasma FFA prevent glucose uptake by muscle [30, 31] and could therefore explain the increase in blood glucose levels in the INS trial. In support of this suggestion, reductions in plasma FFA through treatment with acipimox have been shown to decrease fat oxidation, increase carbohydrate oxidation, and lower blood glucose during moderate intensity exercise in T2D patients [32]. Patients with T2D have impaired ability to oxidize muscle glycogen during exercise [19], and the lower plasma glucose availability in the INS trial perhaps explains the greater increase in FFA in that trial.

Individuals with untreated T2D have increased resting energy expenditure, that is, associated with greater gluconeogenesis [33], but a lower thermic response to food intake [34] in comparison to insulin-sensitive controls. Short-term (8 days) subcutaneous insulin injection is sufficient to lower the resting metabolic rate and increase the thermic response to food intake [35]. Furthermore, 12 months of subcutaneous insulin is also sufficient to reduce the resting metabolic rate and lower hepatic glucose production [36]. The present study extends those findings by reporting a lower resting metabolic rate in response to acute insulin infusion (4 hours).

This study has shown that the infusion of insulin to achieve normoglycaemia in resting T2D subjects maintains blood glucose concentrations at euglycaemic levels during subsequent moderate intensity exercise. Subject numbers were limited due to difficulties of identifying and recruiting patients with nonobese T2D, a cohort that represent 10% of T2D patients, who were able and willing to exercise safely. Further studies of larger cohorts are warranted to determine if these results are extended to larger populations. Glucose-lowering medication was stopped 24 hours before study visits. Although this study shows that hypoglycaemia was not present during exercise, the risk would be greater following habitual T2D medication, particularly sulfonylureas and insulin. Further studies are required to elucidate the mechanisms underlying changes in metabolic physiology following normalization of blood glucose in T2D by glucose-lowering medications.

## 5. Conclusions

These data demonstrate that short-duration continuous exercise of moderate intensity is safe when nonobese patients with T2D are exercising following insulin-induced restoration of normoglycaemia. Furthermore, patients with T2D display similar metabolic responses to exercise in the normoglycaemic or hyperglycaemic state. Given the wealth of studies investigating the metabolic responses to exercise and health benefits in obese type 2 diabetes patients (for review see [37]), the data from the present study highlight the need for further research to investigate the potential differences between obese and nonobese patients with T2D in the efficacy of exercise in disease management.

## Figures and Tables

**Table 1:** Subject demographics and inclusion and exclusion criteria for subjects.

Subject demographics	
Age	56.2 ± 2.7 years
Gender distribution	5 male and 1 female participants
Height (m)	1.64 ± 0.04
Weight (kg)	66.7 ± 6.0
BMI (kg/m^2^)	24.5 ± 1.5
Time since diagnosis (years)	7.8 ± 1.4
Diabetes treatment	Metformin (*n* = 6)
HbA1c (%)/mmol/mol	9.4 ± 0.3/78.9 ± 3.8
Fasting blood glucose (mmol/l)	11.3 ± 0.6
Predicted RMR (Schofield equations) [21] (MJ/d)	6.36 ± 0.31
Calculated RMR (MJ/d)	5.31 ± 1.15
VO_2 peak_ (ml/kg/min)	28.7 (82 ± 4% of the predicted values for healthy individuals of similar age)
	
Inclusion criteria	Exclusion criteria
Type 2 diabetes diagnosed >2 yrs before consent	History of cardiac disease
Age 40–69 years inclusive	History of cerebrovascular events or transient ischemic episodes
BMI: <30 kg/m^2^	History of intermittent claudication
Suboptimal glycaemic control: HbA1c > 8% (64 mmol/mol)	Significant hypertension defined as a systolic BP > 170 mmHg and/or diastolic BP > 95 mmHg
Evidence of recent, regular moderate physical activity (PAL of 1.6–1.7) [22]	Any other disease likely to affect the ability to exercise including arthritis and respiratory disease
Normal resting 12-lead ECG	Any cardiorespiratory drugs other than thiazide diuretics, aspirin, ACE-inhibitors, and statins
No significant ECG changes or chest pain during a Bruce protocol exercise ECG to stage III with a normal physiological response to exercise	Secondary complications: any diabetic retinopathy other than mild background retinopathy, nephropathy (proteinuria on >1 occasion or raised creatinine), and sensory neuropathy

Data are presented as mean ± SEM.

**Table 2:** Physiological and metabolic responses to the infusion of insulin (INS) and saline (CON) in patients with T2D whilst resting for four hours.

		Baseline	60 mins	120 mins	180 mins	240 mins
EE	CON	4.29 ± 0.28	4.57 ± 0.41	4.28 ± 0.26	4.29 ± 0.35	4.42 ± 0.33
	INS	4.01 ± 0.24	3.88 ± 0.14	3.91 ± 0.21^∗^	3.78 ± 0.24	3.90 ± 0.16
RER	CON	0.89 ± 0.04	0.80 ± 0.05	0.83 ± 0.02	0.82 ± 0.02	0.85 ± 0.02
	INS	0.89 ± 0.04	0.94 ± 0.02^∗^	0.94 ± 0.02^∗∗^	0.92 ± 0.04	0.91 ± 0.02
CHO ox	CON	0.17 ± 0.03	0.09 ± 0.03	0.12 ± 0.01	0.10 ± 0.02	0.13 ± 0.02
	INS	0.16 ± 0.03	0.20 ± 0.02^∗^	0.19 ± 0.02^∗∗^	0.16 ± 0.03	0.17 ± 0.02
FAT ox	CON	0.04 ± 0.01	0.09 ± 0.02	0.06 ± 0.01	0.07 ± 0.01	0.06 ± 0.01
	INS	0.04 ± 0.01	0.02 ± 0.01^∗^	0.02 ± 0.01^∗∗^	0.03 ± 0.01	0.03 ± 0.01

Data represent *n* = 6, and values are means ± SEM for energy expenditure (EE) in kJ/min, respiratory exchange ratio (RER), carbohydrate oxidation (CHO ox), and fat oxidation (FAT ox) in g/min. Star symbols denote a significant difference between CON and INS (^∗^*P* < 0.05, ^∗∗^*P* < 0.01).

**Table 3:** Physiological and metabolic responses to submaximal cycling following infusion with insulin (INS) or saline (CON) in patients with T2D.

	O_2_ uptake (ml/kg/min)	RER	CHO ox (g/min)	Fat ox (g/min)	Heart rate (beats/min)	RPE (6–20)
CON	14.1 ± 1.1	0.86 ± 0.02	0.80 ± 0.11	0.17 ± 0.04	124 ± 6	11.6 ± 0.6
INS	14.1 ± 1.5	0.74 ± 0.07	0.74 ± 0.07	0.18 ± 0.03	118 ± 7	11.2 ± 0.9

Values represent *n* = 6 and are displayed as mean ± SEM for 30 min of exercise. RER denotes respiratory exchange ratio; CHO ox denotes carbohydrate oxidation rate; fat ox denotes fat oxidation.
